# Development and psychometric evaluation of the Self-Regulated Nursing Care Scale (SRNCS) among Turkish nurses

**DOI:** 10.1186/s12912-026-05140-7

**Published:** 2026-07-28

**Authors:** Zahide Akeren, İhsan Akeren, Hafsa Kubra Isik

**Affiliations:** 1 Department of Nursing, Faculty of Health Sciences, Bayburt University, Bayburt, 69000 Turkey; 2 Department of Psychological Counseling and Guidance, Faculty of Education, Bayburt University, Bayburt, 69000 Turkey; 3 Department of Midwifery, Faculty of Health Sciences, Atatürk University, Erzurum, 25240 Turkey

**Keywords:** Nursing care, Self-regulation, Self-regulated learning, Validity, Reliability

## Abstract

**Background:**

Self-regulation refers to individuals’ ability to control themselves, manage their behaviours and learn from their environment. Self-regulated learning skills may support nurses in providing effective care. This study aimed to develop and psychometrically evaluate the Self-Regulated Nursing Care Scale (SRNCS) for assessing nurses’ self-regulated nursing care skills based on Zimmerman’s Self-Regulated Learning Model (Z’SLM) and the relevant literature.

**Methods:**

The planning, implementation and evaluation components of nursing care were considered in relation to the forethought, performance and self-reflection phases of Z’SLM. Data were collected online between November 2022 and January 2023 from nurses working in state hospitals. Content validity was assessed through expert review. Exploratory factor analysis (EFA) and confirmatory factor analysis (CFA) were performed using data from 201 and 239 nurses, respectively. Criterion validity and reliability were also examined.

**Results:**

EFA yielded a 16-item, three-dimensional structure comprising planning, implementation, and evaluation and explaining 63.9% of the total variance. CFA supported this three-factor structure in an independent sample. Criterion validity was supported by correlations with the Counselling Skills in Nurses Scale. Cronbach’s alpha was 0.95 for the overall scale and 0.81, 0.87, and 0.93 for the planning, implementation, and evaluation dimensions, respectively. The test-retest reliability coefficient was 0.77.

**Conclusion:**

The findings support the validity and reliability of the Turkish form of the SRNCS for assessing self-regulated care skills among nurses providing inpatient care. The scale may be used in research and clinical settings to assess planning, implementation and evaluation dimensions of self-regulated nursing care. Future studies may examine relationships between SRNCS scores and care-related outcomes.

**Supplementary Information:**

The online version contains supplementary material available at https://doi.org/10.1186/s12912-026-05140-7.

## Background

Since people are the focus of the profession of nursing, care is the basis of this profession. Care is not peculiar to the nursing profession, but unique to nursing. The concept of care in nursing is handled in quite different ways, and many definitions have been made [1]. According to Virginia Henderson (1955), nursing care is “helping to meet the basic needs of the patient by considering physical, psychological, cultural, intellectual, and social aspects” [2]. Watson stated that care is a science, and the process is the essence and representation of nursing. Watson also said, care is the fifth dimension that should come after the concepts of health, nursing, environment, and the individual, which are the basis of the profession of nursing, and emphasised the importance of interaction in this process [3]. While the nursing care process conventionally consisted of three stages as diagnosis, planning, and implementation in the early 1950s, the recent, generally adopted version of the process has five steps, and these are data collection, diagnosis, planning, implementation, and evaluation. This process ensures that all nursing services are provided within a certain system. While undertaking diagnostic tasks, evidence-based care planning, implementation, and evaluation, the process is practiced within the framework of the scientific method using a systematic and analytical approach, rather than an intuitive one [4]. Nursing care is shaped by individual, social, organisational, and professional factors.

As medical technology develops, the responsibilities of nurses increase as the number of patients with serious and complex health problems increases. The changing roles and responsibilities of nurses have led to their need to be competent in problem-solving and decision-making to provide individualised and accurate care. Additionally, this situation has made it necessary for nurses to have the knowledge and skills related to the profession required by professional care, be open to change, be committed to the ethical values required by the profession, follow scientific developments related to the profession, and take responsibility [5]. A nurse who fulfils these requirements can provide effective and qualified care. Effective and qualified nursing care contributes to patient recovery, patient satisfaction, and the improvement of health service quality [6].

In this context, it is important for nurses to recognise themselves, manage their professional development, learn independently, and adopt approaches that facilitate the achievement of care-related goals [7]. One of the approaches that can be adopted for this purpose is self-regulated learning. Self-regulation is the ability to understand and manage one’s own behaviours and reactions. Zimmerman defined self-regulation as cyclical and planned feelings, thoughts, and actions that individuals create for themselves to achieve their personal goals and modelled self-regulated learning cyclically by dividing it into three stages: forethought, performance, and self-reflection [8]. The forethought phase involves preparation before taking action. This stage includes the process of analysing tasks such as goal setting, creating a strategic plan, and self-motivational beliefs such as intrinsic motivation, outcome expectations, and self-efficacy. The performance stage includes the process of concentrating, self-control, and observation in practice. In the last stage, the self-reflection stage, the implementation is evaluated, and its failure or success is revealed [9].

Kuiper, Murdock, and Grant designed the theoretical framework of the self-regulated learning model in nursing. It is thought that self-regulation training can develop metacognitive thinking strategies that support the clinical decision-making process and the development of competence in practice [10]. In the study conducted by Kuiper and Pesut to examine the effects of the self-regulated learning theory on the development of the cognitive, metacognitive, and clinical reasoning skills of nurses and student nurses, the authors examined the literature in the fields of social sciences, educational psychology, nursing education, and professional education. They concluded that nurses and nursing students can improve their cognitive and metacognitive skills in clinical contexts by using self-regulated learning strategies [11]. Kurt and Eskimez (2022) collected data using the “Self-Regulated Learning Scale for Clinical Nursing Practices”, which was tested to be valid and reliable in Turkish, in their study that aimed to examine self-regulated learning and factors affecting clinical practice in nursing students. The researchers reported that self-regulated learning rates were higher in female students, first-year students, those who were interested in nursing, and those who saw academicians as role models [12]. In the literature, there are studies that have used the self-regulated learning model before nursing students start their clinical practice [11, 13].

Existing self-regulation instruments in nursing have primarily been developed for nursing students and focus on learning processes during clinical education. These instruments do not directly assess how practising nurses plan, implement, monitor, and evaluate nursing care in inpatient clinical settings. Moreover, instruments assessing related constructs, such as clinical learning, counselling skills, or nursing care quality, do not specifically measure self-regulated nursing care skills within a model-based framework. Therefore, a measurement instrument specifically designed for practising nurses was needed. This study aimed to develop and psychometrically evaluate the SRNCS for assessing self-regulated nursing care skills among nurses providing inpatient care.

## Self-regulation and Zimmerman’s Self-Regulated Learning Model (Z’SLM)

**Fig. 1:**
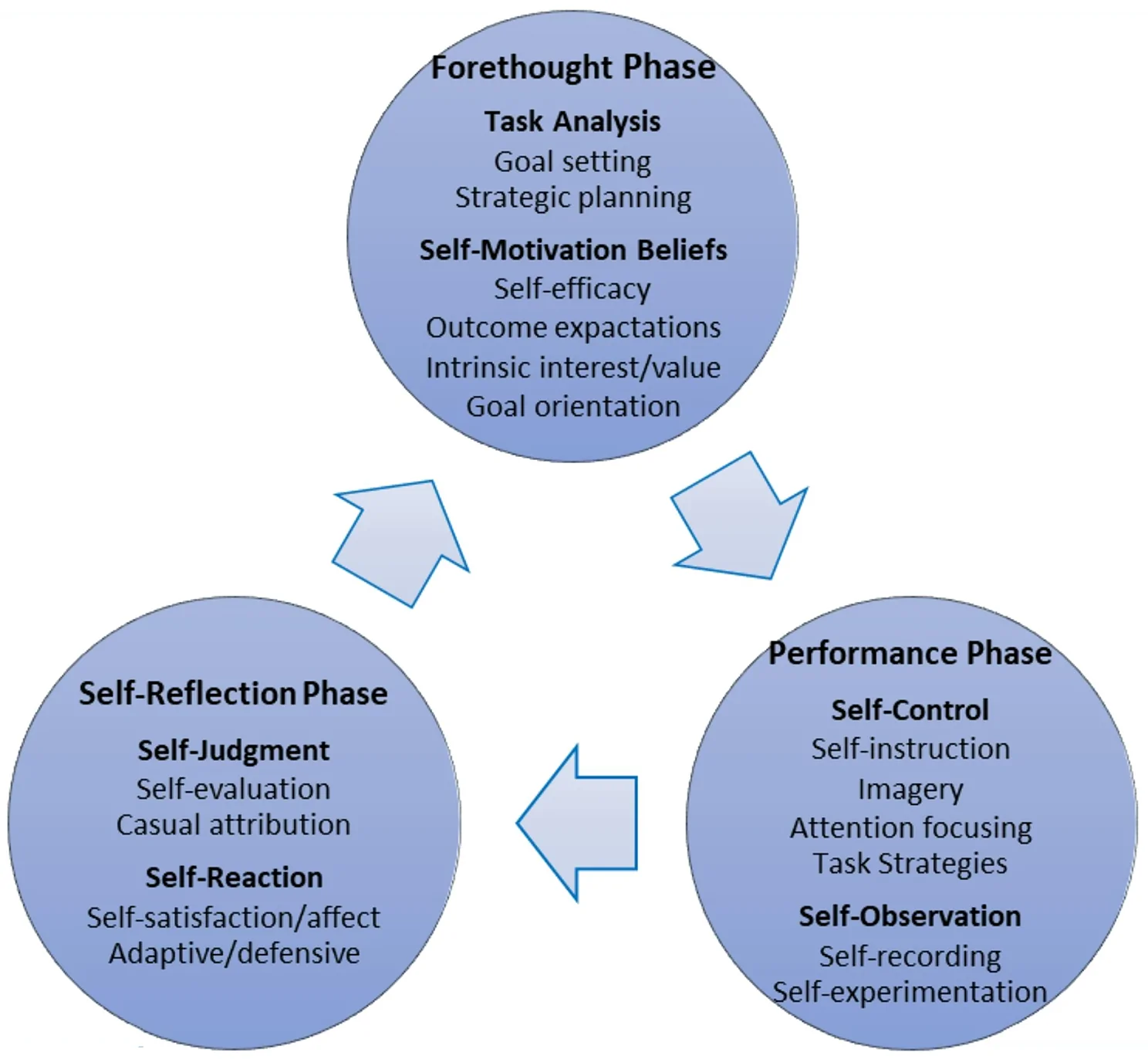
Zimmerman’s self-regulated learning model [8]

Although the concept of self-regulation had been used in different fields of science before, Bandura (1986) was the first to define the concept as we use it today in psychology and educational sciences from the perspective of the Social Cognitive Theory [14]. According to Bandura, self-regulation is an intrinsic quality referring to the extent to which individuals can control themselves cognitively, emotionally and behaviourally and learn from their environment. Because external reward-punishment alone is not determinative on behaviours, individuals continue, change, or stop their behaviours according to the results of their observations [15]. Considering these explanations about the concept, it is understood that self-regulation is also a learning method. As a matter of fact, the emphasis on the learning aspect of the skill has led many researchers (e.g., Boekaerts, Kanfer, Borkowski, Pintrich, Winne) to develop learning models based on self-regulation. One of these models is the self-regulated learning model developed by Zimmerman presented in Fig. 1 [9]. Each model may have shortcomings in terms of their contributions to the understanding of self-regulated learning, but compared to others, Z’SLM addresses self-regulation more comprehensively, formulates it as a cyclical process, takes it out of a limited framework such as learning in the academic field, and emphasises its adaptability to almost every aspect of daily life [16].

According to Zimmerman (2000), the self-regulation cycle of an individual starts with the forethought phase. In this phase, the cognitive, emotional, and behavioural infrastructure of the individual towards the task in hand is formed, and self-reflection has an effect on the decisions made by the individual. The evaluations made by the individual with self-reflection will affect their forethought inferences in later tasks. In this phase, the individual questions the characteristics of the task and their beliefs about the task, and they draw a road map for themselves. The performance phase is the process in which the cognitive, emotional, and behavioural constructs formed by the individual in the forethought phase about the task in hand are put into action. In this phase, the individual focuses on the task, visualises the process in their mind, applies the strategy they have determined, tries different strategies when necessary, and records what they do in the entire process. Self-reflection is the phase involving self-judgement and self-reaction skills in which the individual evaluates their performance and to what extent they have achieved the goals set in the forethought phase [17]. In this phase, the individual compares their performance to the target they have set and determines to what extent they have achieved it. According to the results of this evaluation, the individual will start the cycle of goal setting, planning, and performance again based on their inferences in the forethought phase for the next task or in the repetition of the same task [8].

## Method

### Sample

An a priori power analysis was conducted for the correlation analysis used to examine criterion validity. Since the Self-Regulated Nursing Care Scale (SRNCS) and the Counselling Skills in Nurses Scale (CSNS) measure related but different constructs, a positive but modest correlation was expected between them. Therefore, a one-tailed test and an expected positive correlation of 0.25 were used. As no previous study had provided a directly comparable empirical estimate of the relationship between these constructs, this value was chosen as a modest, conservative expected correlation, rather than being derived from a specific prior study. With α = 0.05 and power = 0.95, the minimum required sample size was calculated as 168 participants. The adequacy of the EFA and CFA sample sizes was evaluated separately based on the recommendations for factor analysis.

Data for the EFA were collected from nurses working in four state hospitals in Erzurum. Data for the CFA were collected from nurses working in three state hospitals in Trabzon and one state hospital in Bayburt. Separate samples were used for EFA and CFA to determine whether the structure identified through EFA could be confirmed in an independent dataset [23]. The nurses recruited from Bayburt constituted a subsample of the CFA group, and the test-retest sample was selected from this group. The study did not have a predefined sampling frame, and a complete list of the nurses who met the inclusion criteria in the participating hospitals was not available. Therefore, the total number of eligible nurses, the number of nurses who received the online form, and the response rate could not be determined. Convenience sampling was used, and nurses who met the inclusion criteria and could be reached during the data collection period were invited to participate.

Fabrigar and Wegener (2011) supported the inclusion of at least 10 participants per parameter in EFA, but they reported that 200 participants would be sufficient in general [18]. Regarding CFA, Kline (2023) stated that 200 participants would be sufficient in most models [19]. The inclusion criteria were being a nurse who had been working for at least one year, actively providing direct care to inpatients in clinical units, and voluntarily agreeing to participate in the study. Nurses working in outpatient examination and treatment units were excluded because the scale was designed to assess self-regulated care skills in nurses providing inpatient care.

## Scale development

### Validity

Garson (2013) stated that a scale is valid when it actually measures the concept intended to be measured without confusing it with anything else and mentioned three forms of validity [20]. These are content validity, construct validity, and criterion validity. The validity analysis steps performed for the scale developed in this study are presented below.

### Content validity

The scale development process started with the examination of self-regulated learning models. For this purpose, self-regulated learning models developed by researchers such as Boekaerts, Kanfer, Borkowski, Pintrich, Winne, and Zimmerman based on the Social Cognitive Theory were identified [8]. Z’SLM was preferred because it divides self-regulation into three cyclical phases as forethought, performance, and self-reflection and explains them clearly. Thus, the theoretical framework of the study was selected as Z’SLM. The initial item pool was created by the research team based on Z’SLM, the relevant literature, and the previous clinical nursing experience of a nursing academician in the research team. Nurses from the target population were not involved in the initial item-generation process. Then, 27 items were created for the planning, implementation, and evaluation dimensions of self-regulated nursing care skills, and the response options were determined as 1 = not at all suitable for me, 2 = not suitable for me, 3 = undecided, 4 = suitable for me, and 5 = completely suitable for me.

The seven disciplinary experts’ evaluations were obtained during the same review round. Once these evaluations had been completed, the form was reviewed by the Turkish Language and Literature expert to ensure linguistic clarity and comprehensibility. Therefore, all eight evaluations were conducted within the same content-validity stage, even though the language review was the last step. The content validity analysis was based on the Davis (1992) criteria [21]. The experts were asked to score each item as 1 = not appropriate, 2 = somewhat appropriate, 3 = quite appropriate, or 4 = appropriate. They were also asked to provide reasons and recommendations for any item that they did not score as 4. All expert ratings were combined in a single form. In calculating the item-level content validity index (I-CVI), ratings of 3 and 4 were accepted as agreement, and the number of experts who rated an item as 3 or 4 was divided by the total number of experts. The I-CVI values of the retained items were either 0.875 or 1.00.

One item had an I-CVI value of 0.625, as five of the eight experts rated it as 4 and three rated it as 1 or 2. This item was related to helping colleagues. In addition to having an I-CVI value below the criterion of 0.80, the experts considered the item to be outside the conceptual scope of self-regulated learning. One expert with doctoral-level expertise in self-regulation stated that, while seeking help can be considered within the scope of self-regulation, helping others is not. Other experts also indicated that this item exceeded the scope of self-regulation. Therefore, this item was removed from the form. No wording or content changes were made to the remaining items following the expert evaluation. Since the retained items were not revised, they were not submitted for a second expert evaluation. Thus, the 26-item draft scale was finalised. The I-CVI values for all original items are presented in Supplementary Table S1.

After the expert evaluation, the 26-item draft form was evaluated by 20 nurses working in state hospitals in two provinces in Central Anatolia who met the inclusion criteria and were actively providing nursing care. This preliminary evaluation was conducted before the main data collection. These 20 nurses consisted of 13 women and 7 men, and they were not included in the subsequent EFA or CFA samples. Thirteen nurses were interviewed face to face. They were given the printed draft form and asked to read and respond to each item. We then asked them what they thought about each item, whether the statements corresponded to their professional practices, and whether the items adequately covered the nursing care practices they performed. Three nurses received the draft form by e-mail and submitted their written comments on the form in person. The remaining four nurses received the draft form via a mobile messaging application, and their feedback was obtained through telephone interviews and recorded in writing by the researchers. All nurses stated that the 26 items reflected different aspects of their professional nursing practices, although the relevance of some practices varied according to the units in which they worked. Therefore, no item was removed or revised after this preliminary evaluation stage.

The item pool was developed and administered in Turkish. Following the psychometric analyses, the final 16-item scale was translated into English by the research team, and the English wording was reviewed through a professional proofreading service. The original Turkish form of the SRNCS is provided in Supplementary Table S2.

### Data collection and analysis

Data were collected between November 2022 and January 2023, after ethical approval had been obtained. Before data collection, the researchers contacted nurses working in state hospitals and informed them about the aim of the study, ethical approval, and voluntary participation. The online questionnaire link was shared with nurses who agreed to participate. The participants were asked to complete the form through Google Forms outside working hours. No item in the form was set as mandatory, and the participants were allowed to leave items unanswered. For EFA, 222 forms were obtained, and for CFA, 244 forms were obtained. Before the analyses, the data were screened for missing values and outliers. For the correlation analyses, the normality of the planning, implementation, evaluation, SRNCS total, and CSNS total score distributions was evaluated using skewness and kurtosis coefficients, histograms, and Q-Q plots. In the EFA dataset, 21 forms were excluded, including 13 forms with outlier values and 8 forms with more than two missing items. In the CFA dataset, 5 forms were excluded for the same reasons. Therefore, the analyses were conducted with 201 nurses for EFA and 239 nurses for CFA. For retained CFA cases containing one or two missing item responses, missing data were handled using pairwise deletion. EFA and CFA were used to test construct validity, and correlation analyses were used to test criterion validity. Criterion validity was examined using the EFA dataset because both the SRNCS and CSNS were administered to the nurses included in this sample (*N* = 201). Cronbach’s alpha and test-retest analyses were used to examine reliability. For test-retest reliability, nurses in Bayburt who were included in the CFA sample, were accessible for follow-up, and agreed to be contacted again were asked to provide an alias for matching. Initially, 48 nurses agreed to participate in the retest. Of these, 39 were reached 6 to 9 weeks after the first administration. Four forms were excluded because their aliases could not be matched with the first administration. Therefore, the test-retest analysis was conducted with 35 nurses. Pearson correlation analysis was used to examine the relationship between the first and second administrations. Confirmatory factor analysis was conducted using JASP version 0.95.4. Because the items had a five-point ordinal response format, the weighted least squares mean- and variance-adjusted (WLSMV) estimator was used. No residual covariances or other post hoc model modifications were specified in the final first-order CFA model.

### Data collection instruments

The Counselling Skills in Nurses Scale (CSNS) was developed by Avcı and Kumcağız (2019) [22]. The scale, which aims to measure the counselling skills of nurses, consists of one dimension and 10 items. It was reported that the scale was valid based on its EFA and CFA results, its Cronbach’s alpha coefficient was calculated as 0.88, its split-half reliability coefficient was calculated as 0.86, and the scale was found reliable. CSNS was used in this study to test criterion validity, as it addresses patient-centred professional behaviours, such as respecting patients, listening to their concerns, monitoring progress in the care process, consulting experienced staff when necessary, encouraging patients and maintaining calm, positive communication. While these behaviours are conceptually related to self-regulated nursing care skills, they are not identical.

## Results

The descriptive characteristics of the EFA, CFA, and test-retest samples are presented in Table 1.

**Table 1:** Descriptive characteristics of the EFA, CFA, and test-retest samples

Variable	EFA (*n* = 201)	CFA (*n* = 239)	Test-retest (*n* = 35)
Age, mean ± SD	30.92 ± 5.89	31.86 ± 7.03	33.40 ± 7.24
Age range	21–46	21–56	23–45
Professional experience, mean ± SD	6.98 ± 4.73	7.88 ± 6.14	10.17 ± 6.85
Professional experience range	1–19	1–32	1–23
Female, n (%)	177 (88.1)	208 (87.0)	28 (80.0)
Male, n (%)	24 (11.9)	31 (13.0)	7 (20.0)

Note. Age and professional experience are presented as mean ± SD; sex as n (%). The test-retest group was a subsample of the CFA group. EFA: exploratory factor analysis; CFA: confirmatory factor analysis

### Construct validity

The data collected from 201 nurses were included in the EFA to test the construct validity of the scale. Before conducting the EFA, the suitability of the sample and data for factor analysis was tested. According to the results of these tests, the Kaiser-Meyer-Olkin (KMO) sampling adequacy coefficient was 0.93, and Bartlett’s Test of Sphericity was significant, χ²(120) = 2332.24, *p* < 0.001. The direct Oblimin rotation technique with Kaiser normalisation was employed due to the theoretical expectation of correlation between the planning, implementation, and evaluation dimensions. Principal axis factoring was chosen as the factor extraction method because the aim of the analysis was to identify latent constructs based on the common variance shared by the items, rather than reducing the observed variables to components. The number of factors expected to be extracted was determined as three based on the forethought, performance, and self-reflection phases of Z’SLM, which constituted the theoretical framework for the item development process. These phases were represented in the scale as planning, implementation, and evaluation, respectively. This three-factor structure was based on Z’SLM and did not refer to the five-stage nursing process. The item factor load criterion was determined as a minimum value of 0.30. During EFA, items with factor loadings below 0.30 or cross-loadings with a difference smaller than 0.10 were removed one by one. After each item removal, EFA was repeated. In total, 10 items were removed from the 26-item draft form. In the final analysis, a three-factor 16-item construct explaining 63.872% of the total variance in the measured variable was obtained. The factors and item-total score correlation coefficients of the identified construct are presented in Table 2.

**Table 2:** Factor loadings and item-total score correlations of the SRNCS (*N* = 201)

Self-regulated nursing care scale (SRNCS)	Planning	Implementation	Evaluation	Item-total correlation	X̄	S. d.
1. I identify the patient’s care needs.	0.486			0.56	4.33	0.65
2. I plan the application steps before I start giving care to the patient.	0.673			0.65	4.33	0.55
3. I envision all steps of the care I will give to the patient.	0.626			0.74	4.28	0.64
4. I observe the patient’s verbal/nonverbal reactions during care practices.		0.791		0.68	4.43	0.56
5. I inquire about the suitability of the care service I apply to the plan.		0.775		0.72	4.25	0.62
6. I make the necessary improvements while giving care to the patient.		0.823		0.68	4.34	0.53
7. I observe the patient for unfavourable situations (side effects/allergic reactions) during the provision of care.		0.611		0.74	4.42	0.61
13. I update my knowledge, when necessary, based on my care experience.			0.564	0.77	4.36	0.59
15. I question how well the care practice serves my purpose.			0.777	0.71	4.18	0.70
16. I question the adequacy of the care I implement.			0.837	0.71	4.26	0.63
17. I question my deficiencies in the patient care process.			0.828	0.79	4.33	0.61
18. I evaluate the quality and outcomes of the care I provide.			0.831	0.82	4.31	0.61
21. I review the application steps again after the implementation of the care practice.			0.712	0.73	4.17	0.70
23. When the patient’s care process ends, I review my roles.			0.758	0.64	4.12	0.89
24. In individualised care practices, I develop different perspectives if necessary.			0.697	0.71	4.17	0.72
26. I try to learn new care practices.			0.583	0.75	4.36	0.66

Note. Item numbers refer to the original 26-item draft form after content validity assessment. Extraction method: principal axis factoring; rotation method: direct Oblimin with Kaiser normalisation. The factors are presented in theoretical order as Planning, Implementation, and Evaluation. Only primary factor loadings are presented. The final three-factor structure explained 63.872% of the total variance. SRNCS: Self-Regulated Nursing Care Scale; X̄: mean; S. d.: standard deviation

Table 2 shows that a three-factor construct was obtained. Based on the original draft item numbers presented in Table 2, items 1, 2, and 3 constituted the planning dimension; items 4, 5, 6, and 7 constituted the implementation dimension; and items 13, 15, 16, 17, 18, 21, 23, 24, and 26 constituted the evaluation dimension. The final 16-item SRNCS is scored on a 5-point Likert scale, and there are no reverse-coded items. The theoretical score ranges are 3–15 for planning, 4–20 for implementation, and 9–45 for evaluation. No cut-off points were established for these dimension scores; therefore, they should be interpreted as continuous scores. In accordance with the theoretically defined and empirically supported three-dimensional structure of the scale, the dimension scores should primarily be calculated and interpreted separately. Higher scores indicate higher self-regulated nursing care skills in the corresponding dimension. A total score may also be calculated as a supplementary continuous indicator; however, no cut-off point was established for the total score, and the primary interpretation of the SRNCS should be based on the planning, implementation, and evaluation dimension scores.

The factor labels were determined by considering both the item content and the phases of Z’SLM. The items collected under the first factor reflected nurses’ identification of care needs and mental preparation before providing care, corresponding to the forethought phase of Z’SLM. The items under the second factor reflected monitoring the care process, assessing its consistency with the plan, and making necessary improvements during care, corresponding to the performance phase. The items under the third factor reflected nurses’ evaluation of the care provided, its outcomes, their own competence, and their roles, corresponding to the self-reflection phase [9]. Accordingly, the three dimensions were named *planning*, *implementation*, and *evaluation*.

The difference in the numbers of items collected under the three factors may be related to the cyclical role of evaluation skills in self-regulation. According to Zimmerman [8], the individual who comes to the forethought phase considers the results of the self-reflection phase and starts the cycle of re-planning and implementation based on these inferences in the next task or in the repetition of the same task. Therefore, the collection of more items under the evaluation factor than under the planning and implementation factors was considered to be consistent with the role of self-reflection in the cyclical structure of Z’SLM.

**Fig. 2:**
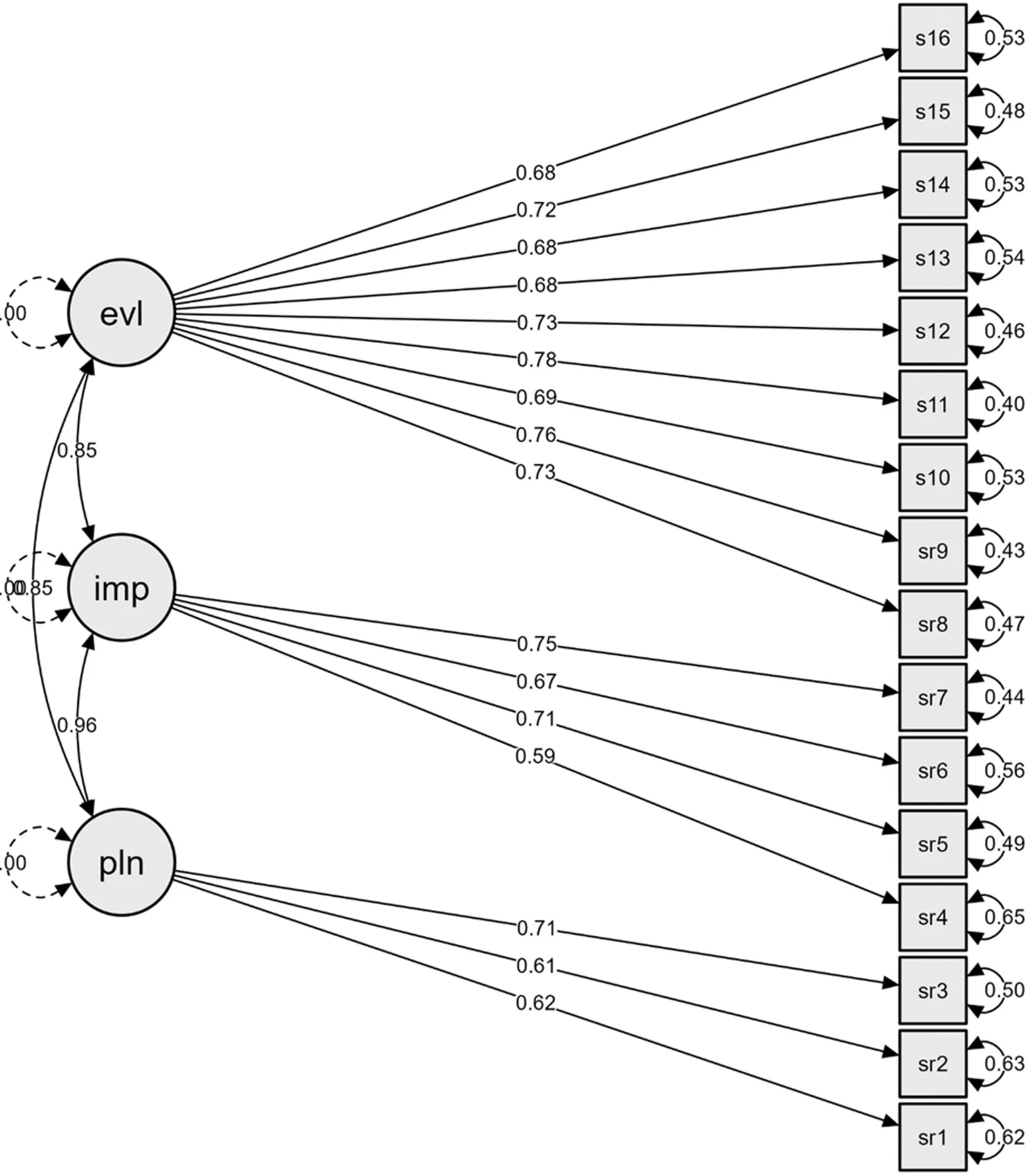
SRNCS first-order CFA results (*N* = 239)

After the construct of a scale is identified, CFA is used to test whether the proposed structure is confirmed in a different dataset [32]. In CFA, model fit is evaluated using several indices, including the chi-square/degrees of freedom ratio (χ²/df), root mean square error of approximation (RMSEA), standardised root mean square residual (SRMR), goodness-of-fit index (GFI), comparative fit index (CFI), normed fit index (NFI), Tucker-Lewis index (TLI), relative fit index (RFI), and incremental fit index (IFI) [33]. In the present study, the first-order CFA supported the three-dimensional structure of the SRNCS, which was consistent with its theoretically defined dimensions and the EFA results [34]. The overall model fit was evaluated by considering the fit indices collectively rather than relying on a single index [35]. The model demonstrated acceptable overall fit: χ²/df = 1.82, RMSEA = 0.059, SRMR = 0.039, GFI = 0.994, CFI = 0.953, TLI = 0.945, NFI = 0.903, RFI = 0.885, and IFI = 0.954. The standardised factor loadings ranged from 0.59 to 0.78 and were statistically significant. Although the correlations among planning, implementation, and evaluation were high, the three dimensions were retained because they corresponded to the forethought, performance, and self-reflection phases of Z’SLM (Fig. 2).

### Criterion validity

The construct validity of the developed scale was supported by determining and verifying its three-factor structure. In the next step, to ensure criterion validity, the SRNCS scores of the participants and their CSNS scores measuring counselling skills were compared. After the final 16-item structure of the SRNCS was obtained through EFA, the factor and total scores of the retained items were calculated. Criterion validity was then examined by analysing the correlations between these SRNCS scores and CSNS total scores. The CFA results obtained for the CSNS showed a χ²/df (88.376/35) value of 2.53, and the model was significant (*p* < 0.001). The other fit indices were also acceptable, including GFI and CFI values above 0.90, RMSEA = 0.072, root mean square residual (RMR) = 0.06, and SRMR = 0.05, and the factor load values of the items ranged between 0.55 and 0.75. In the reliability analysis, the internal consistency coefficient of the scale was identified as 0.88, and its split-half reliability coefficient was 0.84. These results showed that CSNS could be used to test the criterion validity of SRNCS. Before the correlation analyses, the normality of the score distributions of the measurement instruments was tested. In addition, the relationships between the factor and total SRNCS scores and CSNS total scores were examined. The results are presented in Table 3.

**Table 3:** Distribution of measurement scores and correlations with CSNS total scores (*N* = 201)

Variables	Min.	Max.	Mean	S. d.	Skewness	Kurtosis	*r* with CSNS Total
Planning	7.00	15.00	12.94	1.56	-0.45	0.75	0.40**
Implementation	11.00	20.00	17.45	1.97	-0.24	-0.30	0.49**
Evaluation	18.00	45.00	38.28	4.92	-0.49	0.77	0.45**
SRNCS total	40.00	80.00	68.67	7.72	-0.37	0.46	0.50**
CSNS total	30.00	50.00	44.00	4.36	-0.24	-0.53	-

Note. r values indicate Pearson correlation coefficients with CSNS total scores. **p < 0.001. SRNCS: Self-Regulated Nursing Care Scale; CSNS: Counselling Skills in Nurses Scale; S. d.: standard deviation

Skewness and kurtosis coefficients between − 2 and + 2 are considered to indicate an approximately normal distribution [24]. As shown in Table 3, the skewness and kurtosis values for the planning, implementation, evaluation, SRNCS total, and CSNS total scores were within this range. Visual inspection of the histograms indicated no marked skewness, and the points in the Q-Q plots approximately followed the reference lines. After the data-screening and exclusion procedures, no remaining extreme outliers were observed in the score distributions used for the correlation analyses. These findings supported the use of Pearson correlation analysis. The SRNCS dimension and total scores showed moderate positive correlations with CSNS total scores, supporting the criterion validity of the SRNCS.

### Reliability

Reliability is the replicability of the results of a measurement tool. Considering that replicability is a scientific method, researchers need to prove that their instruments are reliable [20].

Using the CFA dataset (*N* = 239), Cronbach’s alpha was 0.95 for the overall scale, 0.81 for the planning dimension, 0.87 for the implementation dimension, and 0.93 for the evaluation dimension. Test-retest reliability was examined with 35 nurses. Pearson correlation analysis showed a significant positive correlation between the first and second administrations of the SRNCS (*r* = 0.77, t(33) = 6.93, *p* < 0.001). These coefficients were above the accepted criterion of 0.70, supporting the reliability of the SRNCS [25].

## Discussion

This study developed and tested the psychometric properties of the SRNCS to assess nurses’ self-regulated care skills. The scale was based on Zimmerman’s cyclical model of self-regulation, which includes forethought, performance, and self-reflection phases [8]. In the context of nursing care, these phases were represented by planning, implementation, and evaluation. Nurses are expected to use skills such as decision-making, problem-solving, monitoring, and self-evaluation during care practices. Moral courage has also been discussed as an important aspect of nursing science and professional practice [26]. Self-regulated learning has been reported to support the development of decision-making, monitoring, and self-evaluation skills [27]. Instruments developed specifically to assess nursing care quality address a related but distinct measurement domain [28]. Therefore, the SRNCS was developed as a model-based tool for assessing self-regulated nursing care skills rather than as a direct measure of nursing care quality.

In scale development studies, reliability, content validity, construct validity, and criterion validity are tested. Content validity is related to the extent to which the data collection tool covers the concept, theory, or variable to be analysed and whether it actually measures it [29]. This form of validity is used to evaluate the suitability of the items in the form for the purpose of the intended measurement [30]. In this study, content validity was examined using the Davis technique [21]. The I-CVI values calculated according to this technique indicated that expert agreement was achieved for the retained items.

Factor analyses are used to test construct validity, which is among powerful parameters in determining validity. In factor analyses, it is aimed to reduce the dataset and make it easier to understand by combining items that measure the same quality [31]. Additionally, sample size also affects factor analysis results. It was reported that the sample size of a study should be tested before performing a factor analysis. The KMO test is used to assess sampling adequacy, with values closer to 1 indicating greater suitability for factor analysis and values below 0.50 generally considered unacceptable [31]. A higher KMO value indicates that the dataset is more appropriate for factor analysis [31]. Bartlett’s Test of Sphericity was also used to evaluate whether the correlation matrix was suitable for factorisation, and a statistically significant result (*p* < 0.05) indicates suitability [31]. In this study, the KMO coefficient was 0.93, and Bartlett’s Test of Sphericity was significant, χ²(120) = 2332.24, *p* < 0.001. These findings indicated that the data were highly suitable for factor analysis.

In our study, the item factor load values ranged between 0.486 and 0.837, and 63.872% of the total variance in the measured variable was explained. Accordingly, the scale items provided construct validity.

After the construct of a scale is identified, CFA is used to test whether the proposed structure is confirmed in a different dataset [32]. In CFA, model fit is evaluated using several indices, including χ²/df, RMSEA, SRMR, GFI, CFI, NFI, TLI, RFI, and IFI [33]. In the present study, the first-order CFA supported the three-dimensional structure of the SRNCS, which was consistent with its theoretically defined dimensions and the EFA results [34]. The overall model fit was evaluated by considering the fit indices collectively rather than relying on a single index [35]. The model demonstrated acceptable overall fit: χ²/df = 1.82, RMSEA = 0.059, SRMR = 0.039, GFI = 0.994, CFI = 0.953, TLI = 0.945, NFI = 0.903, RFI = 0.885, and IFI = 0.954. The standardised factor loadings ranged from 0.59 to 0.78 and were statistically significant. Although the correlations among planning, implementation, and evaluation were high, the three dimensions were retained because they corresponded to the forethought, performance, and self-reflection phases of Z’SLM.

In criterion validity, the relationship between a newly developed measurement instrument and another valid and reliable instrument assessing a conceptually related construct is evaluated [20]. In this study, the CSNS was used to assess criterion validity. The planning, implementation, and evaluation scores showed moderate positive correlations with CSNS total scores (*r* = 0.40, *r* = 0.49, and *r* = 0.45, respectively; all *p* < 0.001). For completeness, the SRNCS total score was also moderately and positively correlated with the CSNS total score (*r* = 0.50, *p* < 0.001). However, consistent with the confirmed three-dimensional structure of the SRNCS, the primary interpretation of the instrument should be based on the planning, implementation, and evaluation dimension scores. These findings supported the criterion-related validity of the SRNCS.

One of the main features sought in a measurement tool is its reliability [36]. Test-retest analysis examines the consistency of responses obtained from the same participants across two administrations. A coefficient between 0.70 and 0.89 is generally considered high, whereas a coefficient between 0.90 and 1.00 is considered very high [29]. Cronbach’s alpha is used to evaluate the internal consistency of items intended to assess the same construct [36], and values above 0.70 or 0.80 are generally considered acceptable [29, 37]. The test-retest coefficient of a measurement instrument should also be at least 0.70 [25]. In this study, the test-retest reliability coefficient of the SRNCS was 0.77. Cronbach’s alpha was 0.95 for the overall scale and 0.81, 0.87, and 0.93 for the planning, implementation, and evaluation dimensions, respectively. These findings supported the internal consistency and temporal stability of the SRNCS.

## Conclusion

In this study, the SRNCS was developed to assess nurses’ self-regulated care skills. The scale has no reverse-coded items, and higher scores on each dimension indicate higher self-regulated nursing care skills in the corresponding area. The findings support the validity and reliability of the Turkish form of the SRNCS among nurses providing inpatient care. The scale may be used in research, clinical practice, nursing education, and nursing management to assess self-regulated care skills across the planning, implementation, and evaluation dimensions. Future studies should examine the psychometric properties of the SRNCS in different nursing populations and investigate its relationships with professional development, clinical decision-making, care-related outcomes, and organisational variables.

### Limitations and future research

The data of this study were collected from nurses working in state hospitals in several provinces and were based on self-reports. Since convenience sampling was used, the findings should be interpreted within the characteristics of the study sample. The high correlations among the three dimensions, particularly between planning and implementation, suggest that their discriminant validity should be examined further in independent samples. Nurses from the target population were not involved in the initial item-generation stage. Although the draft form was subsequently evaluated by 20 nurses, future scale-refinement studies may involve practising nurses directly in qualitative item-generation procedures. Future studies may test the psychometric properties of the SRNCS with larger and more diverse nurse samples from different regions and healthcare institutions. It is also recommended to examine the relationships between nurses’ self-regulated care scores and patient-related outcomes, such as satisfaction with care and perceptions of nursing care. Sociodemographic and professional factors related to nurses’ self-regulated care skills may also be examined in future studies.

## Supplementary Information

Below is the link to the electronic supplementary material.

Supplementary Material 1

## Data Availability

The datasets used and/or analysed during the current study are available from the corresponding author on reasonable request.

## Declarations

### Method suitability

All methods were carried out in accordance with the relevant directives and regulations.
