# Supplementary material for: Development and psychometric evaluation of the Self-Regulated Nursing Care Scale (SRNCS) among Turkish nurses
Source: BMC Nurs. 2026 Jul 28;25:938. doi: 10.1186/s12912-026-05140-7 (PMC13644263; doi:10.1186/s12912-026-05140-7)
Supplement: Supplementary file 1 [file 12912_2026_5140_MOESM1_ESM.docx]

**Supplementary Table S1. Item-level content validity index (I-CVI) results for the original item pool**

Note. Ratings of 3 (quite appropriate) and 4 (appropriate) were accepted as expert agreement according to the Davis technique. I-CVI was calculated as the number of experts rating an item as 3 or 4 divided by the total number of experts (N = 8). Items 1-26 were retained after content validity evaluation and constituted the 26-item draft form. Item 27 was removed after expert review and is presented at the end of the table.

| **Original item no.** | **Turkish item** | **English item** | **Experts rating 1 or 2** | **Experts rating 3** | **Experts rating 4** | **Agreement count (3+4)** | **I-CVI** | **Decision/status** |
| --- | --- | --- | --- | --- | --- | --- | --- | --- |
| 1 | Hastanın bakım ihtiyaçlarını belirlerim. | I identify the patient's care needs. | 0 | 1 | 7 | 8 | 1.00 | Final 16-item SRNCS |
| 2 | Hastaya bakım vermeye başlamadan önce uygulama basamaklarını planlarım. | I plan the application steps before I start giving care to the patient. | 0 | 1 | 7 | 8 | 1.00 | Final 16-item SRNCS |
| 3 | Hastaya vereceğim bakımın bütün basamaklarını zihnimde canlandırırım. | I envision all steps of the care I will give to the patient. | 0 | 1 | 7 | 8 | 1.00 | Final 16-item SRNCS |
| 4 | Bakım uygulaması sırasında hastanın sözlü/sözsüz tepkilerini gözlemlerim. | I observe the patient's verbal/nonverbal reactions during care practices. | 0 | 1 | 7 | 8 | 1.00 | Final 16-item SRNCS |
| 5 | Verdiğim bakım hizmetinin plana uygunluğunu sorgularım. | I inquire about the suitability of the care service I apply to the plan. | 0 | 1 | 7 | 8 | 1.00 | Final 16-item SRNCS |
| 6 | Bakım uygulaması sırasında gerekli iyileştirmeleri yaparım. | I make the necessary improvements while giving care to the patient. | 0 | 1 | 7 | 8 | 1.00 | Final 16-item SRNCS |
| 7 | Bakım uygulaması sonrası hastayı istenmeyen durumlar konusunda (yan etki/alerjik reaksiyon) gözlemlerim. | I observe the patient for unfavourable situations (side effects/allergic reactions) during the provision of care. | 1 | 2 | 5 | 7 | 0.875 | Final 16-item SRNCS |
| 8 | Hastada istenmeyen durumlarla karşılaştığımda (yan etki/alerjik reaksiyon) süreci soğukkanlılıkla yürütürüm. | When I encounter unfavourable situations in the patient (side effects/allergic reactions), I manage the process calmly. | 1 | 1 | 6 | 7 | 0.875 | Removed during EFA |
| 9 | Bakım uygulamalarında yenilikçi yaklaşımları deneyince motive olurum. | I am motivated when I try innovative approaches in care practices. | 1 | 2 | 5 | 7 | 0.875 | Removed during EFA |
| 10 | Hasta bakımında tamamlayıcı uygulamaları bilirim (ağrı gidermede dikkati başka yöne çekme, masaj gibi). | I know complementary practices in patient care (such as distraction for pain relief and massage). | 1 | 1 | 6 | 7 | 0.875 | Removed during EFA |
| 11 | Çalışma arkadaşlarımla farklı bakım yöntemleri hakkında bilgi alışverişinde bulunurum. | I exchange information with my colleagues about different care methods. | 0 | 2 | 6 | 8 | 1.00 | Removed during EFA |
| 12 | Bakım uygulamasında çalışma arkadaşlarımdan gerektiğinde yardım alırım. | I seek help from my colleagues, when necessary, in care practices. | 1 | 2 | 5 | 7 | 0.875 | Removed during EFA |
| 13 | Bakım deneyimlerime göre gerektiğinde bilgilerimi güncellerim. | I update my knowledge, when necessary, based on my care experience. | 0 | 1 | 7 | 8 | 1.00 | Final 16-item SRNCS |
| 14 | Bakım uygulamasında gereken sorumluluğu üstlenirim. | I take the necessary responsibility in care practices. | 1 | 1 | 6 | 7 | 0.875 | Removed during EFA |
| 15 | Bakım uygulamasının amacıma ne kadar hizmet ettiğini sorgularım. | I question how well the care practice serves my purpose. | 0 | 1 | 7 | 8 | 1.00 | Final 16-item SRNCS |
| 16 | Uyguladığım bakımın yeterliliğini sorgularım. | I question the adequacy of the care I implement. | 0 | 2 | 6 | 8 | 1.00 | Final 16-item SRNCS |
| 17 | Hasta bakım sürecinde eksikliklerimi sorgularım. | I question my deficiencies in the patient care process. | 0 | 1 | 7 | 8 | 1.00 | Final 16-item SRNCS |
| 18 | Uyguladığım bakımın kalitesini ve sonuçlarını değerlendiririm. | I evaluate the quality and outcomes of the care I provide. | 0 | 1 | 7 | 8 | 1.00 | Final 16-item SRNCS |
| 19 | Bakım uygulaması sırasında hastanın sözlü/sözsüz geri bildirimlerine dikkat ederim. | I pay attention to the patient's verbal/nonverbal feedback during care practices. | 1 | 2 | 5 | 7 | 0.875 | Removed during EFA |
| 20 | Bakım uygulaması sonrasında hastanın sözlü/sözsüz geri bildirimlerini değerlendiririm. | I evaluate the patient's verbal/nonverbal feedback after care practices. | 1 | 1 | 6 | 7 | 0.875 | Removed during EFA |
| 21 | Bakım uygulaması sonrasında uygulama basamaklarını tekrar gözden geçiririm. | I review the application steps again after the implementation of the care practice. | 0 | 1 | 7 | 8 | 1.00 | Final 16-item SRNCS |
| 22 | Bakım uygulamasının hastada olumlu etki göstermesini beklerim. | I expect the care practice to have a positive effect on the patient. | 1 | 2 | 5 | 7 | 0.875 | Removed during EFA |
| 23 | Hastanın bakımı sonlandığında kendi rolümü sorgularım. | When the patient's care process ends, I review my roles. | 0 | 1 | 7 | 8 | 1.00 | Final 16-item SRNCS |
| 24 | Bireyselleştirilmiş bakım uygulamasında gerekirse farklı bakış açıları geliştiririm. | In individualised care practices, I develop different perspectives if necessary. | 0 | 2 | 6 | 8 | 1.00 | Final 16-item SRNCS |
| 25 | Bakım uygulamalarında kendimi yeterli hissederim. | I feel competent in care practices. | 1 | 1 | 6 | 7 | 0.875 | Removed during EFA |
| 26 | Yeni bakım uygulamalarını öğrenmeye çalışırım. | I try to learn new care practices. | 0 | 1 | 7 | 8 | 1.00 | Final 16-item SRNCS |
| 27 | Bakım uygulamalarında meslektaşlarıma yardım ederim. | I help my colleagues in care practices. | 3 | 0 | 5 | 5 | 0.625 | Removed after expert review |

Abbreviation. I-CVI: item-level content validity index. EFA: exploratory factor analysis. Item 27 was removed after expert review because its I-CVI value was below .80 and experts considered helping colleagues to be outside the conceptual scope of self-regulated learning. One doctoral-level expert in self-regulation stated that seeking help may be within the scope of self-regulation, whereas helping others is not.

**Supplementary Table S2. Original Turkish form of the Self-Regulated Nursing Care Scale (SRNCS)**

**Öz Düzenlemeli Hemşirelik Bakımı Ölçeği (ÖDHBÖ)**

**Yönerge**

Değerli hemşireler, aşağıdaki form hastalara bakım verirken kullandığınız planlama, uygulama ve değerlendirme becerilerinize ilişkin bazı ifadelerden oluşmaktadır. Bu formda kişisel bilgi istenmemektedir. Bu nedenle samimi ve içten cevaplar vermeniz çok önemlidir. Lütfen her bir ifadeyi dikkatlice okuyunuz ve hastalara bakım verirken gerçekleştirdiğiniz uygulamaları dikkate alarak ifadenin size ne düzeyde uygun olduğunu işaretleyiniz. Her ifade için yalnızca bir seçeneği işaretleyiniz.

Katılımınız için teşekkür ederiz.

**Yanıt seçenekleri:** 1 = Bana hiç uygun değil, 2 = Bana uygun değil, 3 = Kararsızım, 4 = Bana uygun, 5 = Bana tamamen uygun

| **No** | **Madde** | **1** | **2** | **3** | **4** | **5** |
| --- | --- | --- | --- | --- | --- | --- |
| 1 | Hastanın bakım ihtiyaçlarını belirlerim. |  |  |  |  |  |
| 2 | Hastaya bakım vermeye başlamadan önce uygulama basamaklarını planlarım. |  |  |  |  |  |
| 3 | Hastaya vereceğim bakımın bütün basamaklarını zihnimde canlandırırım. |  |  |  |  |  |
| 4 | Bakım uygulaması sırasında hastanın sözlü/sözsüz tepkilerini gözlemlerim. |  |  |  |  |  |
| 5 | Verdiğim bakım hizmetinin plana uygunluğunu sorgularım. |  |  |  |  |  |
| 6 | Bakım uygulaması sırasında gerekli iyileştirmeleri yaparım. |  |  |  |  |  |
| 7 | Bakım uygulaması sonrası hastayı istenmeyen durumlar konusunda (yan etki/alerjik reaksiyon) gözlemlerim. |  |  |  |  |  |
| 8 | Bakım deneyimlerime göre gerektiğinde bilgilerimi güncellerim. |  |  |  |  |  |
| 9 | Bakım uygulamasının amacıma ne kadar hizmet ettiğini sorgularım. |  |  |  |  |  |
| 10 | Uyguladığım bakımın yeterliliğini sorgularım. |  |  |  |  |  |
| 11 | Hasta bakım sürecinde eksikliklerimi sorgularım. |  |  |  |  |  |
| 12 | Uyguladığım bakımın kalitesini ve sonuçlarını değerlendiririm. |  |  |  |  |  |
| 13 | Bakım uygulaması sonrasında uygulama basamaklarını tekrar gözden geçiririm. |  |  |  |  |  |
| 14 | Hastanın bakımı sonlandığında kendi rolümü sorgularım. |  |  |  |  |  |
| 15 | Bireyselleştirilmiş bakım uygulamasında gerekirse farklı bakış açıları geliştiririm. |  |  |  |  |  |
| 16 | Yeni bakım uygulamalarını öğrenmeye çalışırım. |  |  |  |  |  |

Not: Ölçek formunda ters puanlanan madde bulunmamaktadır. 1, 2 ve 3. maddeler Planlama; 4, 5, 6 ve 7. maddeler Uygulama; 8, 9, 10, 11, 12, 13, 14, 15 ve 16. maddeler ise Değerlendirme boyutunu oluşturmaktadır. Planlama boyutundan 3–15, Uygulama boyutundan 4–20 ve Değerlendirme boyutundan 9–45 arasında puan alınabilir. Ölçeğin doğrulanan üç boyutlu yapısı doğrultusunda alt boyut puanları ayrı ayrı hesaplanmalı ve öncelikle bu boyutlar üzerinden yorumlanmalıdır. Alt boyutlar için herhangi bir kesme noktası belirlenmemiştir. Her bir boyutta alınan puanın artması, ilgili alandaki öz düzenlemeli hemşirelik bakımı becerilerinin daha yüksek olduğunu göstermektedir. Ölçek toplam puanı yalnızca tamamlayıcı bir sürekli gösterge olarak hesaplanabilir; toplam puan için herhangi bir kesme noktası belirlenmemiştir ve ölçeğin temel yorumu alt boyut puanlarına dayanmalıdır.
